# Evaluating Health Canada’s Proposed Front-of-Pack Labelling Policy: Burden of Healthcare Use Attributed to Health Canada’s Proposed Front-of-Pack Labelling Policy

**DOI:** 10.23889/ijpds.v9i5.2911

**Published:** 2024-09-18

**Authors:** Alisha Buttar, Mahsa Jessri

**Affiliations:** 1 Food, Nutrition and Health, Faculty of Land and Food Systems, University of British Columbia

The economic burden of disease due to consumption of poor diet costs billions of dollars annually to the Canadian healthcare system. This project is in response to the growing interest in the use of nutrient profiling systems on front-of-package (FOP) nutrition labels in Canada. There is a high demand for simplified healthy eating messaging. Health Canada announced improving food environments is a priority in the Healthy Eating Strategy and will implement the mandatory FOP label policy in 2026. Two Ontario-based studies examined the impacts of health behaviors (smoking, alcohol consumption, poor diet and physical inactivity) attributed to hospital bed-days and costs. They found 36% of hospital use was attributable to all health behaviours resulting in 900,000 bed-days annually. Between 2004-2013, 22% of healthcare costs totalling $89.4 billion were attributable to the four health behaviours. These are likely underestimates as diet was measured based on fruit/vegetables frequency.

The objective is to evaluate Health Canada’s Proposed FOP policy on hospital bed-days.

Detailed dietary data from the nationally representative cross-sectional national survey data Canadian Community Heath Survey-Nutrition 2004 linked to Discharge Abstract Database (2004-14) for hospital use will be used for analysis. Data-driven methodology will be employed and development of multivariable risk models using zero-inflated negative binomial regression with multiple exposure groups to test a dose-response.

Results are undergoing, will be ready September 2024.

This is the first study to analyze Health Canada’s proposed FOP policy related to economic outcomes. These findings will provide evidence-based recommendations to policymakers on nutrition policy.

